# Substernocleidomastoid Muscle Neck Lipoma: An Isolated Case Report

**DOI:** 10.1155/2019/4936357

**Published:** 2019-06-19

**Authors:** Ferdinand Rico, Dustin Hoang, John Lung, Olivia Puccio, Michelle Brito, M. Haris Nazim, Alan Sbar

**Affiliations:** Department of Surgery, Texas Tech University Health Sciences Center, 1400 S Coulter, Amarillo, TX 79106, USA

## Abstract

**Introduction:**

We report this large neck mass, located behind the sternocleidomastoid (SCM) within the posterior cervical space and anterior to the prevertebral fascia. The mass is displacing the carotid sheath and its neurovascular contents medially. It extends almost the whole length of the SCM muscle all the way down to the lung apex.

**Case Presentation:**

A 30-year-old female patient presented to our clinic with a left anterior neck mass for a few months with dysphagia. The lipoma was excised completely along with level II to IV lymphadenectomy. A very small volume chyle leak was noted intraoperatively. The drain was removed 2 weeks later only to recur in one month. A new drain was placed by interventional radiology, and the drainage resolved completely.

**Discussion:**

The patient with mild dysphagia had a lipoma large enough to push vital structures away from their normal anatomical position. Due to the difficult location and the size of the lipoma, a meticulous formal lateral neck dissection was required. A 3D imaging like CT or MRI would be helpful to preoperatively plan the dissection. Substernocleidomastoid neck lipoma in our case is a rare benign tumor that was challenging to excise.

## 1. Introduction

Neck lipomas are rare, slow-growing, benign tumors that can be asymptomatic or cause various neck symptoms and signs that include pain, dysphagia, and hoarseness. Most frequently, the presentation is of an asymptomatic, pain-free, slowly growing mass involving subcutaneous tissue [1]. Many asymptomatic neck lipomas are located along with the subcutaneous tissue space, less commonly in the neck musculature or invading through various vital structures. Lipomas can be deep in cervical tissue and rarely exceed 5 cm in size. They may be located or adjacent to cervical skeletal muscle and bone surfaces [2] . They can vary widely in their origin and distribution [3]. Lipomas are the most common abnormal mass of mesenchymal origin, with 80% of benign lipoma tumors classified as ordinary lipomas and 13% occurring in the head and neck. The subcutaneous layer in the posterior neck is the most common location [4]. Lipomas are often underreported since many are unreported to physicians unless symptomatic due to an anatomical or cosmetic defect. Many asymptomatic neck lipomas are located in the subcutaneous tissue but can also be found within the musculature of the neck or invading through various structures including the larynx [5], the sternocleidomastoid muscle [6], and the carotid sheath [7]. There have been reports of neck lipomas previously [8–15], but few large lipomas extending to the lung apex and requiring carotid sheath dissection [7] have been described and reported in the literature. We report this case of a substernocleidomastoid neck lipoma that extended to the lung apex, abutting on the cervical thoracic duct and the carotid sheath. We describe the careful dissection and removal of the lipoma and discuss postoperative management after neck dissection.

## 2. Case Presentation

**Figure 1:**
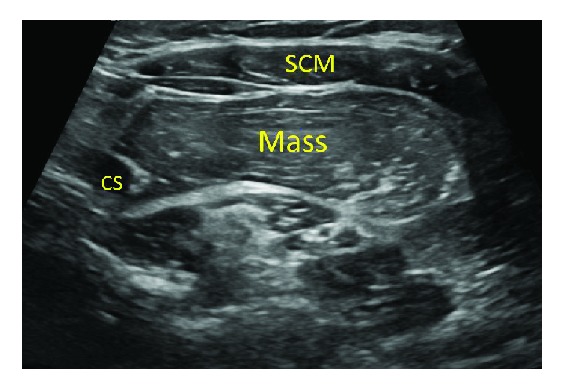
Ultrasound of the soft tissues of the left side of the neck in the area of clinical interest demonstrating a circumscribed solid echogenic mass measuring 6.7 cm × 1.8 cm × 4.8 cm consistent with a lipoma. The mass is displacing the sternocleidomastoid muscle (SCM) and carotid space (CS).

A 30-year-old female patient presented to our clinic with a left neck mass that she noticed a few months prior to clinical evaluation. She had a full range of motion in her neck and rarely complained of pain but did notice a lot of difficulty swallowing. The patient reported the mass has been increasing in size. She denied any fever, chills, nausea, vomiting, redness, or drainage around the mass. The rest of her history was unremarkable to the case. On physical exam, the patient was noted to have a BMI of 43.67 and all vital signs were within normal limits. The physical exam showed a left neck mass with poorly defined borders, nontender, and without inflammatory changes. The patient previously had an ultrasound of the left neck which demonstrated a circumscribed solid echogenic mass measuring 6.7 cm × 1.8 cm × 4.8 cm which corresponded to the palpable abnormality superior to the clavicle. The mass was identified as a lipoma (Figure 1).

**Figure 2:**
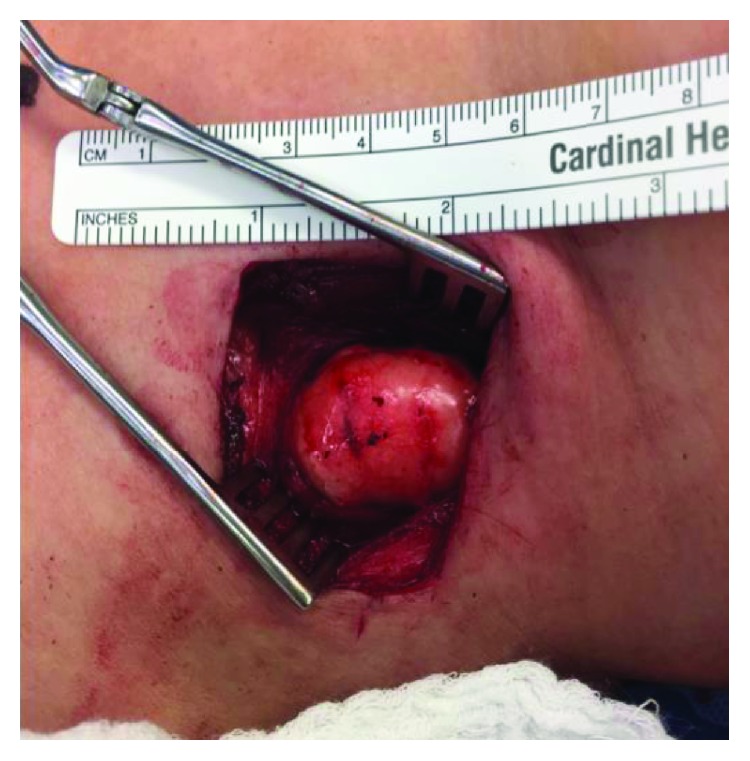
View of lipoma after initial dissection with release of the left investing layer (posterior fascia of the sternocleidomastoid muscle) of the deep cervical fascia.

**Figure 3:**
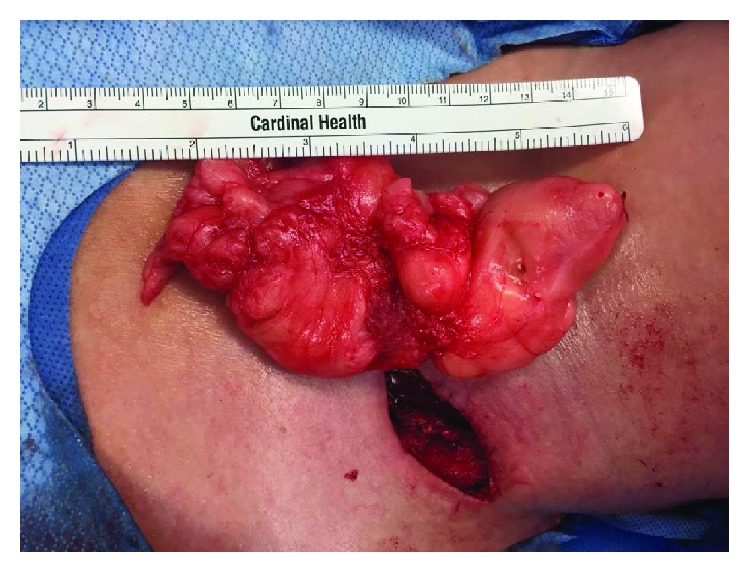
Excised mass-lipoma measuring around 12 × 5 × 2 cm in size, laid over the midanterior neck intraoperatively, with noted left lateral neck incision (*N.B.*: right side of the picture is the patient's head).

A left lateral transverse incision and dissection showed no subcutaneous mass. Intraoperative Doppler showed extreme medial displacement of the carotid sheath vessels. Then, a formal lateral neck dissection released the medial investing fascia of the sternocleidomastoid muscle enabling its further lateral retraction (Figure 2). The mass was located substernocleidomastoid, from the C3 vertebral level down to the lung apex. It was medially displacing and abutting both the carotid sheath and the cervical thoracic duct as it drains into the internal jugular and subclavian vein junction. Subsequent carotid sheath dissection was performed with exposure of the internal jugular vein and common carotid artery at its internal/external branching. Also noted during the lipoma excision were large suspicious lymph nodes in the area posterior to the sternocleidomastoid. A formal left lymphadenectomy at levels II-IV was done. Free lymphatic channels near the apex of the lung and internal jugular vein were noted with small clear to milky fluid exudation. Fibrin glue was sprayed on the chyle leak site. Further examination of the dissection site and the mass itself extracorporeally showed complete removal (Figure 3). A small JP drain was applied. Her postoperative course was uneventful, and the patient was discharged the following day with a drain in place. The pathology report of the soft tissue mass showed mature adipose tissue consistent with a lipoma. The cervical lymph nodes were benign with no atypical lymphoid infiltrates, granulomas, or metastatic disease.

The drain was removed after 2 weeks of follow-up when the drainage stopped only to present later with swelling and erythema surrounding the JP drain site. A CT of the neck showed a fluid collection at the area of the JP drain site measuring 3.7 × 3.1 cm. She underwent IR drainage of the fluid and returned 10 cc of chyle fluid. It drained the same volume amount daily. Fluid culture came back positive for MSSA—methicillin-sensitive *Staphylococcus aureus*. She was discharged 3 days later with a 5-day course of Bactrim. The drain was removed one week after discharge.

## 3. Discussion

Lipomas are slow-growing, benign tumors composed of adipose tissue that can occur anywhere in the body. Approximately 13% are located in the head and neck regions. They are commonly located in the posterior neck in the subcutaneous tissue layer, external to the superficial cervical fascia [14]. This case presents a rare look at a large anterior neck lipoma that compresses and distorts normal neck anatomy.

**Figure 4:**
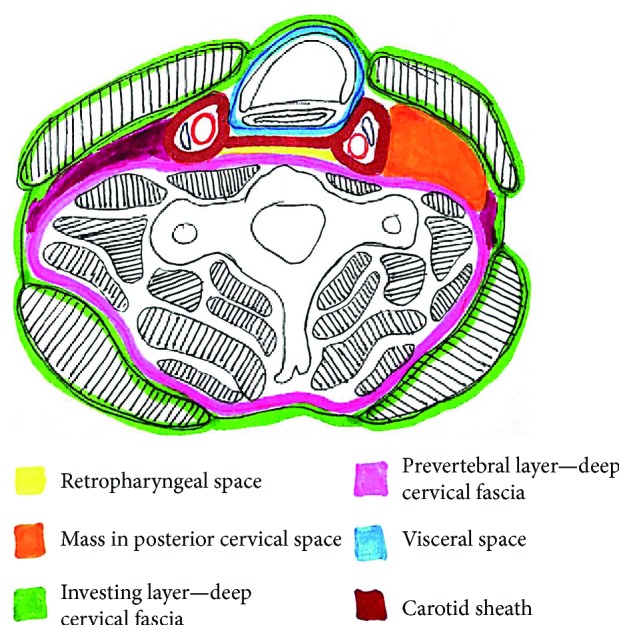
Schematic of sub-SCM location of the mass (orange) that displaced the left SCM muscle. The mass was also noted to medially displace the carotid sheath. Dissection was required through the investing layer of the deep cervical fascia to the prevertebral layer of the deep cervical fascia.

We reviewed the literature of lipomas, specifically on those in the deep anterior neck area. An intramuscular SCM lipoma was noted in the anterior neck area [11]. A deep cervical intramuscular lipoma that caused neck and occipital pain was also reported [15]. Some case reports of large neck lipomas presented asymptomatically while others presented with limited range of motion and neurological signs such as upper limb paresthesia [12–14]. We report this case of a lipoma located at the posterior cervical space. It extremely displaced medially the carotid sheath requiring carotid sheath dissection. It also extended down to the lung apex as well as abutting the thoracic duct with its radicles as it enters the internal jugular and subclavian vein junction. This resulted in a chyle leak during dissection. The sub-SCM location of the mass required a dissection through the investing layer to the prevertebral layer of the deep cervical fascia to reach the mass located in the posterior cervical space (Figure 4). The dissection required lateral displacement of the left platysma and SCM muscles. Thus, the location, size, and abutment of the mass to vital anatomical neck structures required a formal lateral neck dissection to expose the mass. The patient presented in our clinic with a neck mass that appeared as a simple lipoma with a less detailed ultrasound report. A high index of suspicion with regard to the complexity of the neck mass, location, and anatomical contiguity with vital organs is of prime importance in preoperative surgical decision warranting more detailed CT or MRI imaging study.

Formal left level II-IV lymphadenectomy was added in the procedure as it was contiguous with the mass and very well exposed in the dissection. Lymph nodes were sent for formal histopathological evaluation with the mass in the event it turned out to be malignant. When confronted with a complex and complicated lipomatous mass, liposarcoma and Madelung's disease should be included in the differential diagnosis for it can be hard to differentiate clinically. This further stresses the need for histopathologic analysis [16, 17].

The thoracic duct is a major structure on the left side close to the apex of the lung as it exits into the vein junction of internal jugular and subclavian veins. Careful and meticulous dissection is of extreme importance to prevent a chyle leak. Should it happen to be injured, the surgeon should attempt to determine a low or high output level and be able to manage the complication intraoperatively. Cyanoacrylate glue and fibrin glue have been reported to be of value in sealing the leak with good outcomes [18]. Others reported successful surgical repair with an omohyoid muscle flap [19]. Postoperative treatment involves a reduction of fat in the diet, octreotide, and drainage with interventional radiology or endoscopic surgery. When all options have failed, reexploring the surgical site is recommended [18].

Our patient presented with dysphagia with no other neurological signs or complaints of pain. The lack of symptoms reported from the patient suggested that the lipoma would be superficially located and not located substernocleidomastoid. Our patient required an extensive lateral neck and carotid sheath dissection without 3D imaging available to visualize structural abnormalities. Our dissection illustrates the need to appreciate neck anatomy in order to avoid damage to the brachial plexus, the accessory nerve, lymph nodes, and the carotid space (carotid artery, vagus nerve, and internal jugular vein). Our case illustrated the need for a full preoperative workup even on lipomas that appeared clinically benign and demonstrated a good operative dissection plan with a favorable postoperative outcome even without 3D imaging to guide neck dissection.

This case report of a substernocleidomastoid neck lipoma is an isolated one. It is a rare benign tumor that required a formal lateral neck dissection. A preoperative CT scan with IV contrast or an MRI cannot be underemphasized as an aid in delineating the mass with its contiguous vital anatomical structures. We recommend that surgical management of this kind of lipoma, though benign, should be reserved for experienced surgeons well-versed in formal deep neck dissection and capable of managing surgical complications.
